# On the optimization of copy number variations representation in pangenome graphs

**DOI:** 10.3389/fbinf.2026.1811916

**Published:** 2026-05-01

**Authors:** Mirko Coggi, Lorenzo Basile, Beatrice Branchini, Gabriele Amodeo, Guido Walter Di Donato, Marco D. Santambrogio

**Affiliations:** 1 Department of Electronics, Information and Bioengineering, Politecnico di Milano, Milan, Italy; 2 GenoGra s.r.l., Milan, Italy

**Keywords:** copy number variations, genome graphs, genomics, pangenomics, topology optimization

## Abstract

Graph-based pangenome references often misrepresent Copy Number Variations (CNVs) and Variable Number Tandem Repeats (VNTRs) as alternative acyclic paths, which hinders downstream analyses, degrades alignment behavior, and reduces interpretability in graph visualizations. For these reasons, we introduce PANPHORTE, a topology-optimization methodology that detects repeat-driven misrepresentations within superbubbles and rewrites them into structures that more faithfully reflect the underlying biology. Given a pangenome graph annotated with haplotype paths, PANPHORTE identifies repetitive elements inside superbubbles, isolates shared repeat sequences across distinct subpaths, and refactors the graph by splitting nodes and introducing explicit cycles, encoding CNVs and VNTRs without loss of information. We provide a C++ command-line implementation of the proposed specifications, and a complementary pipeline that applies PANPHORTE followed by GFAffix to further reduce redundancy in regions not affected by repeat-induced artifacts. We evaluate PANPHORTE on synthetic and real pangenome graphs, showing reductions in memory footprint of up to 71.69%, improvements in exact read matches of up to 34.4%, and substantially clearer visual identification of repeated loci.

## 1 Introduction

In the last decade, Next-Generation Sequencing (NGS) has dramatically increased the speed and scale at which genomic data can be generated. One of the most impactful developments enabled by NGS is pangenomics, an area of computational genomics that aims to characterize genetic variability across multiple individuals within a species or clade (Eizenga et al., 2020). This paradigm departs from classical reference-based analysis: a single linear reference genome provides only one reference representation of each locus and therefore cannot capture the full extent of population diversity. Evidence from the Human Pangenome Reference Consortium (Wang et al., 2022) shows that dependence on a single reference can introduce reference allele bias, namely the systematic underrepresentation of sequences that diverge from the chosen reference, leading to errors in resequencing and read mapping, particularly for population-specific variation. By contrast, relating an individual genome to a pangenome representation can reduce this bias and recover a broader spectrum of genetic diversity (Eizenga et al., 2020).

**FIGURE 1:**
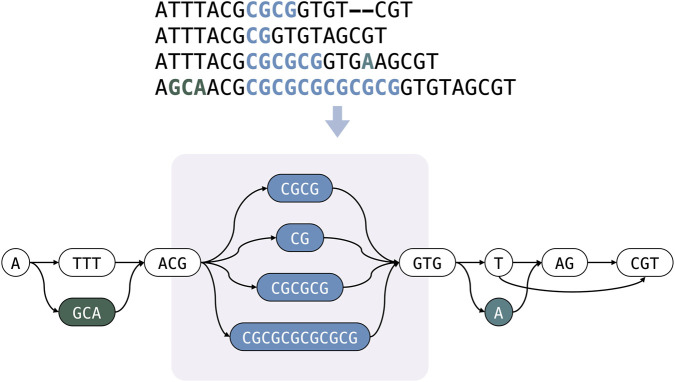
Representing a set of related sequences as a variation graph enables the explicit encoding of SVs that would otherwise be missed or mischaracterized when using a single linear reference genome; nevertheless, in many scenarios, as in the highlighted region, pangenomes are represented as a DAGs in which CNVs are incorrectly modeled as distinct alternative paths, yielding a bifurcating topology rather than a cyclic repeat.

A common and expressive formalism for representing pangenomes is the sequence graph. In this model, genomes or haplotypes are represented as paths (or *walks*) in a directed graph, allowing shared sequences to be represented once while alternative sequence realizations diverge locally, providing a compact representation that can simultaneously accommodate common and rare alleles. Sequence graphs naturally capture Single-Nucleotide Polymorphisms (SNPs), small indels, and large Structural Variants (SVs) by extending the linear reference into a variation graph, as depicted in Figure 1. They can also represent repetitive loci, including Copy Number Variations (CNVs) and Variable Number Tandem Repeats (VNTRs), by introducing cycles over the repeated sequence (Garrison et al., 2018; Paten et al., 2017). Although CNVs and VNTRs are biologically distinct, with the former altering the copy number of a genomic segment and the latter changing the number of short, adjacent repeat units, their graph representation is conceptually similar. Hereafter, we use the term “CNV” to refer broadly to both forms of copy-number change.

Despite their expressive power, pangenome graphs remain challenging to construct in a way that consistently resolves complex SVs (Garrison et al., 2024). CNVs provide a representative example: repetitive structures are frequently misrepresented as large alternative sequences, producing bifurcations rather than the simpler and more faithful cyclic topology expected for a repeat, as in the graph showed in Figure 1. These artefacts are often attributable to the behavior of sequence-to-graph alignment algorithms, which may prioritize local sequence differences over the broader structural context (Coggi et al., 2025). The resulting bifurcations hinder interpretation of the underlying genomic architecture and can complicate downstream analyses such as variant detection and genotyping. Consequently, the biological signature of CNVs may be obscured because the inherent repetitiveness of the locus is not represented explicitly. For instance, if a new sample carries a copy number for a given CNV that is not captured by any of the alternative sequences encoded in the graph, the alignment may fail to match and typically requires additional investigation to interpret the underlying repeat variation. By contrast, a cyclic representation can still yield a valid alignment even when the sample has a different number of repeat units, reducing the problem to estimating whether its copy number differs from those observed in the haplotypes used to construct the graph. A further advantage of representing CNVs as cycles, rather than enumerating them as alternative sequences, is the reduction of redundancy in repetitive elements. This compression of repeated content can substantially reduce the graph’s memory footprint, which is particularly valuable given that pangenome graphs are already large and resource-intensive data structures.

For these reasons, we propose PANPHORTE, an approach that takes as input a pangenome graph, detects repeated regions that are misrepresented as alternative sequences, and refactors them into explicit cyclic structures. By enforcing a topology that reflects the repetitiveness of such loci, PANPHORTE yields a more compact representation of the pangenome reference graph without loss of information, supports more streamlined alignment of new samples carrying CNVs, and facilitates visual recognition of repeated regions. In addition, we release an open-source C++ command-line tool^1^ that implements the PANPHORTE specifications and performs automated topology optimization of the representation of CNVs in pangenome graphs. Furthermore, we propose a PANPHORTE-based pipeline for graph topology optimization. The pipeline, after superbubble identification via BubbleGun (Dabbaghie et al., 2022), invokes PANPHORTE followed by GFAffix (Doerr et al., 2022) to further refine the graph topology, especially in those regions where no repeat-induced misrepresentation is detected. To assess the efficacy of PANPHORTE, we evaluated our tool on both synthetic and real datasets, with the goal of isolating its key benefits under controlled conditions and demonstrating its practical impact on representative pangenomes.

We show that PANPHORTE achieves up to 70% memory reduction and increases the proportion of exact matches by up to 34% when aligning CNV-rich reads to the optimized pangenome graphs. On real datasets, the PANPHORTE-based pipeline yields up to 8.74% memory savings while preserving the information content of the original graph.

This manuscript is an extension of the preliminary work presented at the Conference “Computational Intelligence Methods for Bioinformatics and Biostatistics 2025”.

## 2 Materials and methods

### 2.1 Data

To test our methodology, we rely on both real and synthetic data to better capture the improvements delivered by our approach. Real datasets used in our experiments comprised two complementary resources. First, we analyzed a collection of ten *Escherichia coli* assemblies from the RefSeq repository, downloaded from the NCBI Genomes FTP service as genomic FASTA files. Second, we used 61 assemblies from the Major Histocompatibility Complex (MHC) FASTA dataset which is referenced in the sequence-to-graph alignment literature and associated with the work by Zhang et al. (2022).

These two datasets were selected to probe graph construction on biologically distinct variation regimes: *E. coli* provides a compact bacterial genome where population-scale differences across strains are readily observable, whereas the human MHC is widely recognized as one of the most polymorphic regions of the human genome and is therefore frequently adopted as a stress test and case study in pangenomic method development and evaluation (Chin et al., 2023). In both cases, the downloaded FASTA assemblies were used as input to the Minigraph-Cactus pipeline (Hickey et al., 2024) to construct the corresponding genome graphs. Table 1 reports the two graphs’ main feature.

**TABLE 1:** Metrics extracted from real data graphs. *Edge degree* is calculated as number of *edges* over number of *nodes*. The number of *haplotypes* matches the number of assemblies used to create the graphs. We reported the *longest path* in each graph expressed in Megabases. We counted the number of *bubbles* in each graph and the portion presenting misrepresentation of CNVs (*bubbles with CNVs*). Finally we computed the *average number of paths* and the number of *unique paths* inside each bubble.

Metrics	*E. coli*	MHC
Number of nodes	401,565	186,804
Number of edges	541,091	258,290
Edge degree	1.35	1.38
Number of haplotypes	10	61
Total bp (Mb)	12.01	28.63
Longest path (Mb)	5.22	5.14
Number of bubbles	127,894	54,453
Number of bubble with CNVs	574	724
Average number of paths per bubble	9.98	60.39
Number of unique paths per bubble	2.07	2.18
Average number of nodes per bubble	2.12	2.26
Average number of bp per bubble	60.04	143.19

As for the synthetic data, we constructed a dataset derived from the 25 chromosomes of the T2T-CHM13v2.0 reference genome Nurk et al. (2022). For each chromosome, we simulated CNVs events by randomly selecting an insertion coordinate and introducing a synthetic sequence l repeated w times. This procedure was repeated 30 times per chromosome, generating 30 haplotypes that share the same insertion locus and repeat unit l , while differing in the copy number w , emulating inter-individual copy-number variability at a fixed genomic site. Table 2 reports the distribution of the simulated CNVs events inserted in each graph as a function of the repeat-unit length l and the maximum copy number w . For instance, 40% of the CNVs correspond to repeat units between 2 and 100 bp with up to 100 copies. As the repeat unit becomes longer, events are generated less frequently and with smaller copy numbers, reflecting plausible biological trends while preventing excessive graph growth in the synthetic benchmarks.

**TABLE 2:** Characterization of CNVs inserted in the pangenomes. Each simulated CNV inserts a repeat unit of length l that is repeated w times. Rows define the strata for l and the maximum number of copies $w_{\max}$ ; the percentage indicates the fraction of all simulated CNVs drawn from each stratum.

CNV fraction	Repeat length l (bp)	Max. Number of copies $w_{\max}$
40%	2–100	100
30%	100–1,000	100
20%	1,000–10,000	10
10%	10,000–100,000	10

Using this simulation framework, we generated three sets (Set 1, Set 2, and Set 3), each comprising 30 haplotypes and containing 100, 1,000, and 10,000 CNV events, respectively, distributed across the T2T-CHM13v2.0 reference genome’s chromosomes. For each set, we constructed 25 chromosome-specific pangenome graphs using the first 10 haplotypes. The remaining 20 haplotypes were then fragmented into synthetic reads of length 10 kb and 100 kb for assessing the alignment accuracy before and after applying the correcting tools.

### 2.2 Definitions

First, we define a sequence graph as the triple G=(V,E,σ) , where $V=\left\{v_{1},v_{2},\ldots ,v_{\left|V\right|}\right\}$ is a finite set of nodes, E⊆V×V is a set of directed edges, and σ:V→Σ assigns a string of characters from the alphabet Σ to each node (for genomes Σ={A,C,G,T} ). On a sequence graph, it is possible to define a path sequence. Let the list $\pi =\left(v_{\pi }^{1},v_{\pi }^{2},\ldots ,v_{\pi }^{\left|\pi \right|}\right)$ be a path in G=(V,E,σ) , where each $v_{\pi }^{i}\in V$ and $\left(v_{\pi }^{i},v_{\pi }^{i+1}\right)\in E$ . The path sequence of π is defined as the concatenation of the sequence of strings $\sigma \left(\pi \right)=\sigma \left(v_{\pi }^{1}\right)\;\sigma \left(v_{\pi }^{2}\right)\cdots \sigma \left(v_{\pi }^{\left|\pi \right|}\right)$ . Note that the path π may visit the same node multiple times, if the edges of the graph allows it. The set of paths inside G is represented by P(G) , which is infinite if G contains cycles. In sequence graphs, *haplotypes*, i.e., related genomic sequences, are path sequences. Thus, it is possible to define a finite set of haplotype paths in G as $H\left(G\right)=\left\{\pi_{1},\pi_{2},\ldots ,\pi_{\left|H\right|}\right\}\subseteq P\left(G\right)$ .

We leverage the definition of superbubble reported by Onodera et al. (2013). A subgraph with a source node s∈V and a sink node t∈V is called a *superbubble* b(s,t) in G=(V,E,σ) if the subgraph between s and t is directed and acyclic, and the set of nodes reachable from s coincides with the set of nodes from which t is reachable. Let $B=\left\{b_{1},b_{2},\ldots ,b_{\left|B\right|}\right\}$ be the set of superbubbles in G and b(s,t) a superbubble with entry s and exit t belonging to B . From the set of haplotype paths traversing s and t , i.e., $H_{b}\left(G\right)=\left\{\pi_{i}\in H\left(G\right):s,t\in \pi_{i}\right\}$ , we extract the set of haplotype subpaths confined within b as $H\left(b\right)=\left\{\pi_{b,i}:\pi_{b,i}\subseteq_{o}\pi_{i}\in H_{b}\left(G\right)\;\wedge v_{\pi_{b,i}}^{1}=s\;\wedge v_{\pi_{b,i}}^{\left|\pi_{b,i}\right|}=t\right\}$ . Then, we define in Equation 1 the set of unique subpaths within b as:

$$P_{u}\left(b\right)=\left\{\pi_{b}\in H\;\left(b\right):\forall \;\pi ,\pi^{\prime }\in H\;\left(b\right),\;\sigma \;\left(\pi_{b}^{\prime }\right)=\sigma \;\left(\pi_{b}\right)\Rightarrow \pi_{b}^{\prime }=\pi_{b}\right\}$$ (1)

Moreover, we define a mapping $\mu_{b}:P_{u}\left(b\right)\to H\left(G\right)$ as $\mu_{b}\left(\pi_{b}\right)=\left\{\;\pi_{i}\in H\left(G\right):\pi_{b}\subseteq_{o}\pi_{i}\;\right\}$ , to retain the haplotype paths from which each unique subpath is derived.

We now formalize the notion of repetitive elements along a path. Given a path π∈P(G) , a *repetitive element* occurring on π is a tuple r(π)=(l,w,start,end) , where $l\in \Sigma^{*}$ is a substring of σ(π) , $w\in N^{+}$ denotes the number of consecutive occurrences of l in σ(π) , $start=\left(v_{\mathrm{start}},pos\right)$ consists of a node $v_{\mathrm{start}}\in V$ and a position pos∈N specifying the starting position of the first occurrence of l within $\sigma \left(v_{\mathrm{start}}\right)$ , and $end=\left(v_{\mathrm{end}},pos\right)$ consists of a node $v_{\mathrm{end}}\in V$ and a position pos∈N giving the offset from the end of $\sigma \left(v_{\mathrm{end}}\right)$ to the ending position of the last occurrence of l within $\sigma \left(v_{\mathrm{end}}\right)$ .

The set of repetitive elements identified along π is denoted by $R\left(\pi \right)=\left\{r_{1}\left(\pi \right),\ldots ,r_{\left|R\right|}\left(\pi \right)\right\}$ . For a superbubble b , let $P_{u}\left(b\right)$ be the set of unique subpaths $\pi_{b}$ collected within b ; we then define the family of repetitive-element sets associated with these subpaths as $R_{b}=\left\{\;R\left(\pi_{b}\right):\pi_{b}\in P_{u}\left(b\right)\;\right\}$ . A common repetitive sequence of a superbubble is a sequence l that is shared by at least two repetitive elements drawn from two distinct unique subpaths. The set of all common repetitive sequences of a superbubble is defined in Equation 2 as:

$$L\left(b\right)=\left\{\;l\in \Sigma^{*}:\left|\left\{\pi_{b}\in P_{u}\left(b\right):\left(\exists \;w_{\pi_{b}}>0\;|\;\left(l,w_{\pi_{b}},start,end\right)\in R\left(\pi_{b}\right)\right)\right\}\right|\geq 2\right\}$$ (2)

### 2.3 Methods

In this Section, we present the PANPHORTE methodology for pangenome graph morphology optimization. We first detail its novel algorithm, and, then, we briefly discuss the implementation details of the PANPHORTE optimization pipeline.

#### 2.3.1 Proposed PANPHORTE algorithm

**FIGURE 2:**
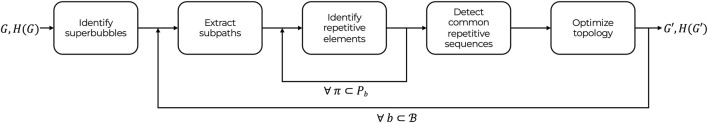
Schematic overview of the PANPHORTE algorithm. Starting from an input pangenome graph G and its haplotype paths H(G) , the method first identifies the set of superbubbles B . For each superbubble b∈B , it extracts the bubble-crossing subpaths and, for every traversal $\pi \in P_{b}$ , detects repetitive elements. Repeats shared by at least two distinct traversals are then consolidated into a set of common repetitive sequences, which guides a local topology rewrite that factors out repetition as an explicit cyclic structure. The process iterates over all superbubbles and returns the optimized graph $G^{\prime }$ together with the updated haplotype paths set $H\left(G^{\prime }\right)$ .

We implemented a path-aware optimization procedure for pangenome graphs that identifies misrepresented repetitive regions within superbubbles and refines the graph topology by converting these regions into cycles. The steps of our algorithm are depicted in Figure 2. Given an input graph G and its associated set of haplotype paths H(G) , PANPHORTE operates in two conceptual stages: (i) it identifies candidate repeat motifs supported by multiple bubble-internal traversals; and (ii) it rewrites each affected superbubble into a compact representation that models repetitions as a cycle, while consistently updating the corresponding haplotype paths. The overall aim is to improve the structural interpretability of repetitive loci and reduce redundancy by replacing multiple alternative bubble-internal paths with a shared repeat unit whose multiplicity is represented through repeated traversal of a self-loop.

**FIGURE 3:**
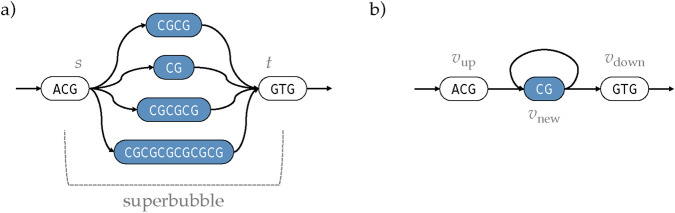
**(a)** In pangenome graphs, CNVs are frequently misrepresented as distinct alternative paths within a superbubble b(s,t) ; **(b)** our graph topology optimization algorithm refactors these structures into cyclic representations, capturing the repetitive nature of CNVs more faithfully within sequence graphs.

The algorithm begins by identifying the collection of superbubbles in G , represented as source–sink pairs (s,t) and stored in B . For each superbubble b∈B (Figure 3a), the pair of endpoints $\left(s_{b},t_{b}\right)$ is obtained. Using the path set H(G) , PANPHORTE then enumerates the set of distinct bubble-crossing traversals induced by the haplotypes. Concretely, it derives the set of *unique subpaths* that traverse b , denoted $P_{u}\left(b\right)$ , and an associated mapping $\mu_{b}$ that links each subpath $\pi_{b}\in P_{u}\left(b\right)$ to its realization in the underlying graph. In practice, each $\pi_{b}$ corresponds to the bubble-internal subsequence extracted from at least one haplotype path; for each such traversal, the corresponding segment sequences are concatenated to form a bubble-internal haplotype sequence. Superbubbles with fewer than two bubble-internal traversals are discarded, since motif selection relies on support across multiple alternative traversals.

For every unique subpath $\pi_{b}\in P_{u}\left(b\right)$ , the algorithm derives its path sequence and extracts its *repetitive elements*, yielding a set $R\left(\pi_{b}\right)$ . Repeats are detected via a deterministic left-to-right scanning strategy designed to emulate non-overlapping repeat discovery. At each position p , the algorithm tests motif lengths ℓ=1,2,… and checks whether the candidate motif s[p:p+ℓ] matches the immediately following substring s[p+ℓ:p+2ℓ] . When the smallest ℓ yielding a match is found, the repeat is extended to its maximal multiplicity k≥2 such that k consecutive copies match. The motif, multiplicity, and position are recorded, and the scan advances by kℓ , ensuring non-overlapping matches. Motifs shorter than or equal to a user-defined threshold are ignored to mitigate low-complexity artefacts. The resulting repeat sets are aggregated at the superbubble level as R(b) .

Once R(b) has been computed, PANPHORTE determines the set of common repetitive sequences l(b) by comparing repetitive elements across distinct unique subpaths and retaining those motifs that occur as repeated substrings in at least two different traversals. only motifs supported by at least two distinct subpaths are eligible. among eligible motifs, PANPHORTE selects a representative motif m∈l(b) by prioritizing maximal motif length (favoring specificity), maximal number of supporting subpaths/haplotypes, and maximal aggregate repeat multiplicity across supporting traversals when explicitly annotated. This selection criterion favors repeat units that are both structurally informative and consistently shared across alternative bubble traversals.

Whenever L(b)≠∅ , PANPHORTE performs a local topological rewrite of the current graph $G^{\prime }$ and the associated haplotypes $H\left(G^{\prime }\right)$ , aimed at factoring out the shared repetition and reducing structural redundancy. given the selected motif m , a new node $v_{\text{new}}$ is introduced with $\sigma \left(v_{\text{new}}\right)=m$ , and a self-edge $\left(v_{\text{new}},v_{\text{new}}\right)$ is added to encode repetition as a cycle that can be traversed multiple times (Figure 3b). For each subpath $\pi_{b}$ that contains an occurrence of m with multiplicity k , PANPHORTE isolates the corresponding repeated region by splitting the occurrence into upstream and downstream boundaries. Specifically, the traversal sequence is decomposed as:

$$h=u\cdot m^{k}\cdot d$$

where u and d denote the upstream and downstream flanking sequences, respectively. Non-empty flanking sequences are materialized as boundary nodes $v_{\text{up}}$ and $v_{\text{down}}$ : $v_{\text{up}}$ preserves the prefix preceding the repetition and inherits the incoming adjacencies of the original start boundary, whereas $v_{\text{down}}$ preserves the suffix following the repetition and inherits the outgoing adjacencies of the original end boundary. When the repeated region begins at the first position of a node (ends at the last position of a node), the corresponding boundary node coincides with the predecessor (successor) along $\pi_{b}$ . The nodes spanning the isolated repeated region, together with their incident edges, are then removed and replaced by new links from $v_{\text{up}}$ to $v_{\text{new}}$ and from $v_{\text{new}}$ to $v_{\text{down}}$ , as illustrated in Figure 3b. Moreover, whenever identical flanking sequences arise across multiple traversals, they are reused rather than duplicated, ensuring that shared flanks do not induce redundant segment proliferation.

At the path level, haplotypes $H\left(G^{\prime }\right)$ supporting the rewritten superbubble are updated to reflect the modified topology. Bubble-internal nodes that previously encoded the haplotype-specific realization of the repeat are removed from the corresponding path, and the sequence of newly introduced elements, i.e., the upstream boundary (when present), k traversals of $v_{\text{new}}$ , and the downstream boundary (when present), is inserted at a consistent position relative to the bubble start endpoint $s_{b}$ . The procedure preserves the invariant that all segments referenced by any walk in $H\left(G^{\prime }\right)$ exist in the segment set of $G^{\prime }$ ; in particular, nodes are removed from the graph only when they are no longer referenced by any haplotype traversal after the update. After processing all b∈B , the algorithm returns the optimized graph $G^{\prime }$ , in which selected repeats within superbubbles are represented explicitly as cyclic structures and in which all affected haplotype walks are rewritten to traverse the new compact representation.

It is important to note that, although PANPHORTE is presented in the context of walk-annotated pangenome graphs, haplotype-path annotations are not required for the core detection of repeat-driven graph structures. Candidate regions are identified from graph topology and from the sequences associated with bubble-internal traversals, whereas haplotype walks become relevant only in the subsequent step that restores consistency between embedded paths and the rewritten graph. As a consequence, PANPHORTE can also be applied to graphs lacking explicit haplotype annotations, such as those produced by assembly-only pipelines, in which case the topological optimization can still be performed but no walk-reconciliation step is needed.

### 2.4 PANPHORTE C++ tool

We developed a standalone command-line C++ tool that implements the PANPHORTE optimization methodology on pangenome graphs. The program takes as input a graph stored in Graphical Fragment Assembly (GFA)^2^ v1.1+ with haplotype paths encoded as walk annotations (W-lines) and a JSON file reporting the positions of the superbubbles in the graph, in terms of source-sink pairs and nodes traversing the superbubble paths. Then, for each superbubble in the JSON, our PANPHORTE tool gathers the set of bubble-internal haplotype traversals by extracting from the GFA walk records the nodes that fall inside the bubble. The nucleotide sequences of these traversals are reconstructed by concatenating the sequences of the corresponding segments. Repeats are detected within each reconstructed traversal using a regex-like approach: a deterministic left-to-right scan designed to report non-overlapping repeated substrings while filtering motifs below a user-defined minimum length, as detailed in Section 2.3. Candidate motifs are pooled across traversals and evaluated according to the PANPHORTE selection logic: only motifs supported by at least two distinct traversals are eligible, and the chosen motif prioritizes longer repeat units and broader cross-traversal support. Once a motif is selected for a superbubble, the tool applies the rewrite scheme described in the previous Section: it introduces a motif segment with a self-loop to represent repetition and, for each supporting traversal, replaces the original bubble-internal nodes with the corresponding upstream flank (if any), the repeated motif, and the downstream flank (if any). The associated walk records, i.e. the haplotype paths, are updated accordingly to ensure consistency between the modified topology and the walk-encoded haplotypes.

To translate the theoretical PANPHORTE procedure into a robust tool implementation that is compatible with downstream tools, we explicitly enforce the same walk-level invariants and consistency requirements described earlier. In particular, after rewriting a superbubble, the affected haplotype paths are updated to reflect the modified topology by removing the original bubble-internal nodes and inserting the newly introduced elements at a fixed position relative to the superbubble entry. It is important to note that the implementation preserves the invariant that every segment referenced by any walk in the output still exists in the segment set: bubble-internal segments are deleted only if, after all walk updates have been applied, they are no longer referenced by any walk. Additionally, to avoid artefacts introduced by repeated application of local rewrites, the implementation suppresses duplicate links so that each edge is emitted uniquely. The resulting output is a GFA graph that retains the original header and metadata, while instantiating the optimized graph $G^{\prime }$ envisioned by the PANPHORTE methodology, where selected repeats in superbubbles are represented as cyclic structures and the corresponding walks traverse the compact representation.

### 2.5 Proposed PANPHORTE-based graph topology optimization pipeline

**FIGURE 4:**
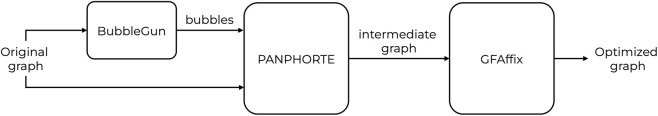
PANPHORTE-based topology optimization pipeline. Given an input pangenome graph, BubbleGun is used to identify superbubbles and export their boundaries. PANPHORTE then refactors repeat-driven superbubbles into explicit cyclic structures and updates the corresponding haplotype walks, producing an intermediate graph. Finally, the intermediate graph is processed with GFAffix to further simplify the topology, yielding the final optimized graph.

Finally, we introduce a practical pipeline for graph topology optimization that combines PANPHORTE with a state-of-the-art graph manipulation tool, namely GFAffix (Figure 4). The pipeline is designed to be both targeted and computationally efficient: rather than rebuilding the entire graph, it focuses refinement on the specific loci where topology is most likely to be suboptimal, particularly in the presence of repeat-driven variation.

The workflow begins with superbubble identification on the input pangenome graph. To this end, we use BubbleGun (Dabbaghie et al., 2022) to compute bubble chains and export superbubble descriptions in JSON format. BubbleGun is well suited to this role because it provides explicit superbubble boundaries and internal node sets, which can be consumed directly to drive local, boundary-aware rewrites without requiring additional graph-specific instrumentation.

Next, our C++ implementation of PANPHORTE is applied to the set of detected superbubbles to optimize their topology, with an emphasis on refactoring repetitive regions that are often misrepresented as acyclic alternatives. This step produces an updated graph in which repeats are modeled through cyclic structure, and in which the walk-encoded haplotypes are rewritten to remain consistent with the modified topology. As a consequence, the optimized graph may exhibit a new superbubble decomposition and an updated set of traversals reflecting the refactored representation.

Although PANPHORTE addresses a broad class of repeat-driven topological artefacts, portions of the graph outside its scope may still benefit from further refinement. This includes, for instance, regions in which no repeat-induced misrepresentation is detected, or cases where the upstream and downstream flanking segments introduced during factorization do not fully resolve into a uniquely simplified local structure. For these reasons, our pipeline applies an additional post-processing step with GFAffix (Doerr et al., 2022), a widely adopted tool for topology simplification in state-of-the-art graph construction workflows, including the one used by the Human Pangenome Reference Consortium (Wang et al., 2022; Liao et al., 2023). Importantly, PANPHORTE and GFAffix operate according to complementary principles, and their combination can therefore yield additive improvements. PANPHORTE targets repetitions by enforcing an explicit cyclic representation within affected superbubbles, whereas GFAffix simplifies graphs by merging node affixes whenever overlap structure permits. Consequently, in repetition-free regions where PANPHORTE performs no modification, GFAffix can still reduce redundancy and streamline the topology. This complementary behavior improves the overall compactness of the graph and can facilitate downstream analyses by reducing unnecessary structural complexity.

In our PANPHORTE optimization pipeline, we rely on BubbleGun v1.2.0 and GFAffix v0.2.1.

## 3 Results

### 3.1 Experimental evaluation on synthetic data

We use the synthetic datasets presented in Section 2.1 to quantify the topology optimization achieved by PANPHORTE in terms of reduced storage requirements for the resulting pangenome graphs. In addition, we also showcase downstream consequences by aligning with GraphAligner (Rautiainen and Marschall, 2017) the simulated reads (with CNVs) to the pangenomes before and after optimization, and comparing the total number of matches to determine how PANPHORTE-based graph refinements affect mapping performance.

#### 3.1.1 Memory footprint evaluation

First, we evaluate the effect of the PANPHORTE methodology on the memory footprint of the resulting pangenome graphs. Table 3 reports the storage occupancy (MB) before and after applying PANPHORTE, together with the corresponding compression rate and percentage change. The results show a clear dependence on CNV burden: memory savings increase as the number of CNVs grows, ranging from a modest effect in the graph built from Set 1 to substantial reductions for CNV-rich graphs (up to approximately 70% for the one built on Set 3). Consistently, the compression rate improves with increasing numbers of inserted CNVs, indicating that explicit cyclic modeling becomes progressively more beneficial as repetitive structure accumulates. Importantly, these gains do not come at the expense of information content: PANPHORTE performs purely topological rewrites that preserve the sequences and walk-encoded haplotypes represented in the original graphs.

**TABLE 3:** Memory footprint of the three sets before and after applying PANPHORTE, memory reduction percentage, and attained compression rate.

Graph	Number of CNVs	Pre [MB]	Post [MB]	% reduction in memory	Compression rate [Pre/Post]
Graph built on set 1	100	3,247	3,077	−5.25%	1.06
Graph built on set 2	1,000	5,124	3,231	−36.94%	1.59
Graph built on set 3	10,000	23,512	6,659	−71.69%	3.53

#### 3.1.2 Alignment evaluation

To quantify the downstream impact of explicitly modeling CNVs as cyclic structures, we aligned the simulated read sets to the corresponding pangenome graphs before and after applying PANPHORTE. Alignment accuracy was measured as the proportion of *exacly aligned* reads, which serves as the primary metric in this experiment. All alignments were performed with GraphAligner (Rautiainen and Marschall, 2020) using its default settings.

Table 4 reports the percentage of perfectly aligned reads for the three datasets, considering both 10kb and 100 kb reads. Across all experiments, PANPHORTE increases the fraction of perfectly matched reads, yielding post-optimization values above 98% in every scenario. The magnitude of the improvement grows with both the CNV burden of the graph and the read length, consistent with the intuition that longer reads and more repetitive loci benefit most from a topology that makes copy-number structure explicit. The most pronounced effect is observed for 100 kb reads aligned to the graph built from Set 3, where the gain in perfect-alignment rate approaches 35%. Overall, these results support the claim that cyclic CNV representations improve sequence-to-graph alignment by providing a structurally faithful model of variable copy number, consequently facilitating exact mappings that can be interpreted by comparison against the walk-encoded haplotype paths.

**TABLE 4:** Percentage of perfectly matched reads, before and after applying PANPHORTE.

Read length	Set	No. of CNV	% Of matches before PANPHORTE	% Of matches after PANPHORTE	Improvement [%]
10k	Set1	100	98.93	99.50	0.57
	Set2	1,000	98.33	99.55	1.22
	Set3	10,000	96.15	99.81	3.66
100k	Set1	100	93.94	98.63	4.69
	Set2	1,000	73.27	98.92	25.65
	Set3	10,000	64.07	98.47	34.40

### 3.2 Experimental evaluation on real data

To assess the practical effectiveness of our methodology, we apply it to the MHC and *E. coli* genome graphs described in Section 2.1. We focus on topology optimization in terms of memory footprint reduction obtained by applying PANPHORTE and, subsequently, GFAffix. To gain more insights with respect to PANPHORTE, we also provide a characterization of the graphs after PANPHORTE’s optimization.

**FIGURE 5:**
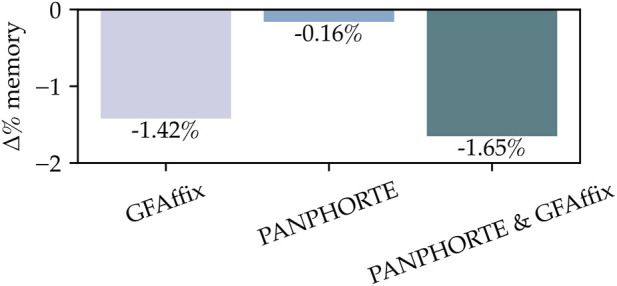
Percentage variation in memory usage in the *E. coli* pangenome, reported as Δ % w.r.t. the original file (negative values indicate a reduction).

For the *E. coli* pangenome graph, Table 5 reports the graph size (MB) under three optimization settings: PANPHORTE only, GFAffix only, and the sequential application of both tools. As expected, PANPHORTE yields a partial reduction because its rewrites are restricted to superbubbles exhibiting repeat-driven structure (0.4% of all superbubbles as reported in Table 1). As reported in Table 6, PANPHORTE is able to optimize the 574 superbubbles with CNVs, identifying repetitions of up to 178 bp with occurrences of up to 9 times. Thus, PANPHORTE yields a reduction in the total number of bps in the graph. From Table 5, we can notice that GFAffix achieves larger savings. This results from the tool’s abitlity to operate globally across the graph. Nevertheless, the combined pipeline attains the largest reduction, decreasing the graph size by up to 0.73 MB. This improvement is not merely additive: the intermediate graph produced by PANPHORTE exposes simplified patterns that GFAffix can more readily exploit, enabling additional affix-merging opportunities. Consistently, the percentage changes compared to the original graph size reported in Figure 5 show that the combined strategy yields the strongest reduction compared to either tool applied in isolation.

**TABLE 5:** Variation in memory usage w.r.t. the original file size after applying GFAffix, PANPHORTE, and their combination (our proposed pipeline) on the *E. coli* pangenome. The combined application achieves the largest reduction.

Applied optimizations	File size [MB]
None (original)	43.62
GFAffix only	43.00
PANPHORTE only	43.55
PANPHORTE and GFAffix	**42.90**

Best in bold.

**TABLE 6:** Characterization of the graphs after applying PANPHORTE. We report the resulting numbers of *nodes* and *edges*, as well as the nodes and edges *added* and *removed* by the rewrite. We also quantify the *graph size* (in Mb) and the reduction in *total base pairs*. Finally, we summarize repeat features by reporting the CNVs length range and mean length, together with the repeat-count range and average occurrence.

Metrics	*E. coli*	MHC
Number of nodes	**402,596 (+0.1%)**	**185,654 (-0.7%)**
Number of edges	**543,152 (+0.3%)**	**256,583 (-0.7%)**
Nodes added	3,728	4,012
Nodes removed	2,697	5,167
Edges added	7,042	8,039
Edges removed	4,982	9,746
**Total bp (Mb)**	**11.84 (-1.5%)**	**27.26 (-5%)**
Net bp saved (Mb)	0.17	1.36
**Length of repetitive elements** (l)	**[2 : 178], avg.: 4.72**	**[2 : 193], avg.: 4.65**
**Number of repetitions** (w)	**[1 : 9], avg: 1.69**	**[1 : 25], avg.: 2.58**

Best in bold.

**FIGURE 6:**
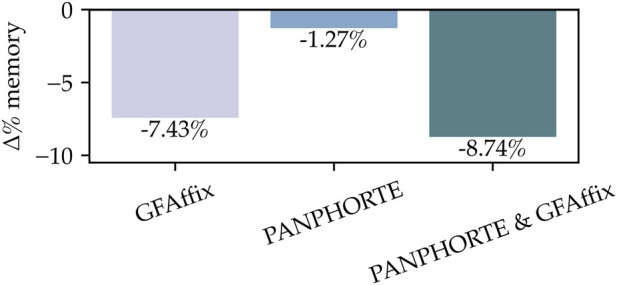
Percentage variation in memory usage in the MHC graph, reported as Δ % w.r.t. the original file (negative values indicate a reduction).

The same trend is observed for the MHC graph. Table 7 reports the graph sizes (MB), while Figure 6 summarizes the corresponding percentage reductions with respect to the original file size. As for the *E. coli* graph, on the one hand, PANPHORTE operates on a minimal part of the superbubbles, around 1.3% (Table 1), delivering a reduced improvement. PANPHORTE is able to identify and optimize repetitions of up to 193 bp and occurring up to 25 times, delivering a total reduction of 1.36 Mb in the graph (Table 6). On the other hand, GFAffix contribution is more considerable, around 7.43%. Nevertheless, also in this setting, the sequential application of PANPHORTE and GFAffix yields the strongest improvement, reducing the graph size by up to 6.88 MB (−8.74%).

**TABLE 7:** Variation in memory usage w.r.t. the original file size after applying GFAffix, PANPHORTE, and their combination (our proposed pipeline) on the MHC graph. The combined application achieves the largest reduction.

Applied optimizations	File size [MB]
None (original)	78.75
GFAffix only	72.90
PANPHORTE only	77.75
PANPHORTE and GFAffix	**71.87**

Best in bold.

It is important to note that the magnitude of these gains depends on structural properties of the input graph, including the number and complexity of superbubbles, the prevalence of repetitive elements, and the extent to which the graph construction already captures repeat structure. In addition, PANPHORTE is most effective when repetitive sequences are embedded in superbubbles and are ambiguously represented, as these can be refactored into cyclic motifs. Conversely, when such configurations are infrequent or already well resolved in the input graph, the scope for improvement is inherently limited. This reflects the deliberately local and biologically targeted scope of PANPHORTE, rather than a limitation of global graph simplification capacity.

## 4 Discussion

This work introduces PANPHORTE, a methodology and accompanying tool for graph topology optimization specifically aimed at improving the representation of repetitive variation in pangenome graphs. By enforcing a topology that makes repeat structure explicit, PANPHORTE produces more compact pangenome graph representations without loss of information. Beyond the reduction in redundancy, the resulting graphs improve the interpretability of repetitive loci by making them structurally apparent.

**FIGURE 7:**
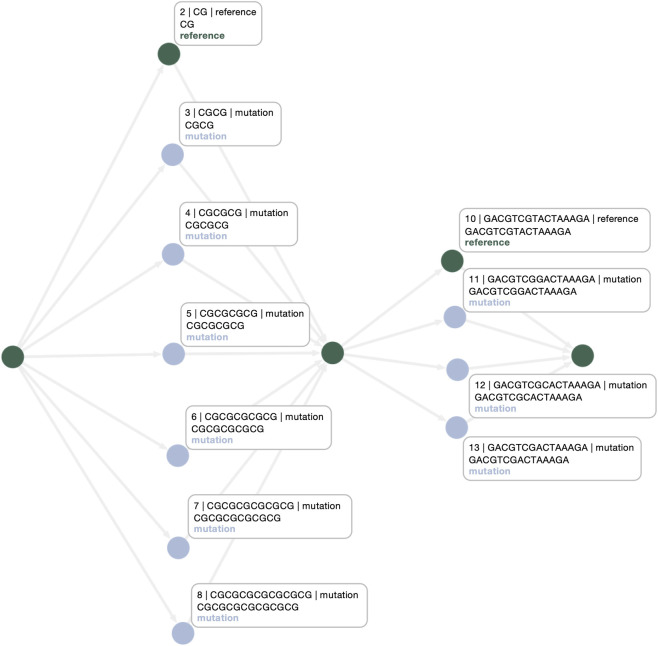
Original example graph containing two superbubbles. The first superbubble encodes alternative haplotype paths with distinct CNVs, whereas the second comprises closely related haplotypes represented as separate paths rather than as localized deviations from the reference.

We illustrate the qualitative benefits of PANPHORTE on synthetic genome graphs by using renderings produced with the GenoGra platform for pangenome analysis GenoGra (2026). Figure 7 reports the layout of the graph used throughout this section: green nodes correspond to the reference, while light-blue nodes belong to alternative haplotypes. The graph contains two superbubbles: the first comprises seven haplotypes carrying CNVs of the segment CG, whereas the second is traversed by four closely related haplotypes that differ only by a small number of SNPs. This compact example allows us to isolate the effect of each stage of the proposed graph-topology optimization pipeline. Also, we argue that this configuration is representative of graphs produced by classical construction approaches, such as Minigraph-Cactus, which may fail to fully resolve specific repetitive patterns and can yield structures analogous to the chosen example.

**FIGURE 8:**
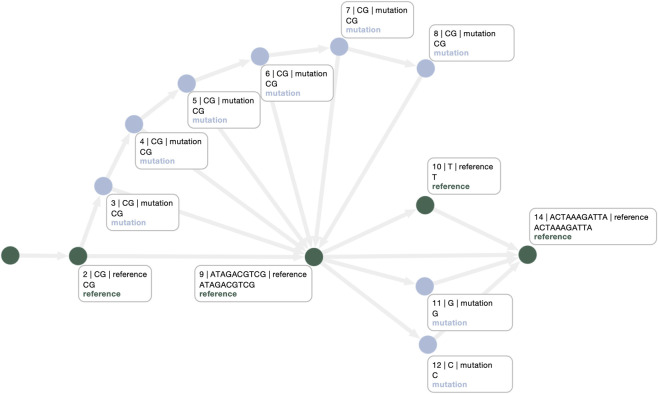
GFAffix-optimized graph. The second superbubble is compacted into SNPs on the reference, whereas the first superbubble retains a suboptimal representation of the CNVs region.

A first optimization step is the application of GFAffix, which reduces sequence redundancy by merging shared affixes across nodes. Figure 8 shows the result of applying GFAffix to the example graph. GFAffix operates globally, rather than targeting specific regions, thus we can see the effects of its optimizations on the entire graph. Specifically, it improves the second superbubble substantially by explicitly modeling SNPs as alternatives. However, the CNVs representation in the first superbubble remains suboptimal, particularly from an alignment perspective. Concretely, when aligning a new sample whose repeat count is not already represented in the graph, the alignment may exhibit an apparent mismatch that requires additional inspection to infer the underlying CNV, complicating downstream analyses.

**FIGURE 9:**
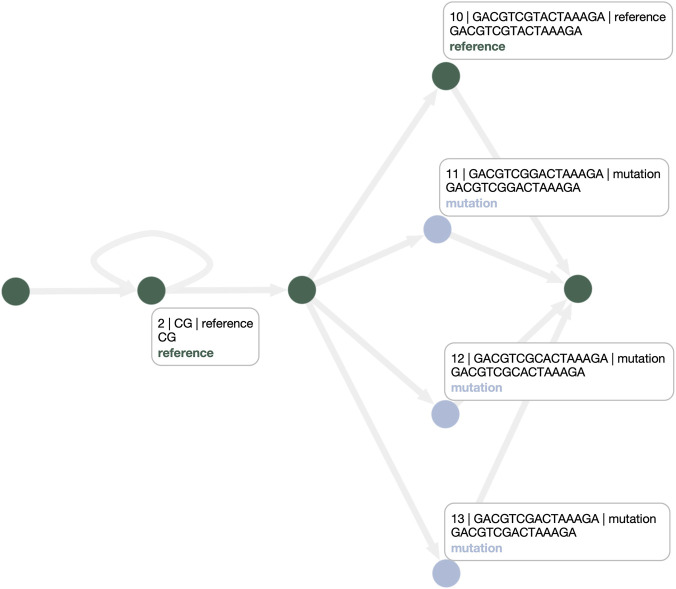
PANPHORTE-optimized graph. The CNVs in the first superbubble are explicitly modeled as a cycle over the reference node, whereas the second superbubble is represented as in the original graph.

**FIGURE 10:**
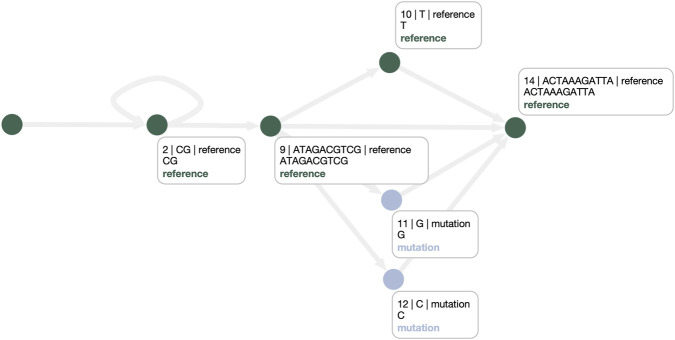
Final graph produced by the proposed pipeline. Both superbubbles are appropriately resolved: the first models CNVs as cycles, whereas the second is compacted into localized variations around the reference path.

This limitation is addressed by the PANPHORTE-optimized graph shown in Figure 9. Here, repetitive elements in the first superbubble are rewritten as an explicit cycle. By transforming ambiguous bubble-like alternatives into cyclic structures, PANPHORTE makes CNVs directly legible from the topology, enabling users to identify both the presence and the distribution of CNVs across the pangenome at a glance. Moreover, an explicit cyclic model improves the alignment of samples with variable repeat counts: any repeat number can be accommodated by traversing the cycle, and inspection of haplotype paths naturally highlights previously unseen CNV multiplicities. It is important to note that PANPHORTE is designed to act only on superbubbles that contain repetitive regions; accordingly, the second superbubble is left unchanged. For this reason, we propose the PANPHORTE-based pipeline that additionally invokes GFAffix to address residual redundancy outside the targeted repetitive structures. Figure 10 shows the combined outcome: PANPHORTE resolves the CNVs as a cycle in the first superbubble, while GFAffix merges common affixes in the second, producing a graph that is both more interpretable in terms of CNVs and SNPs and more compact.

Overall, these examples show that the PANPHORTE topology rewrite makes biologically meaningful variation operationally explicit. It is important to note that PANPHORTE should not be intended as a general-purpose global graph simplification method. Its primary goal is to improve the biological faithfulness of repeat-rich loci by rewriting misrepresented CNV structures into explicit cyclic topologies while preserving the original sequence content and walk-encoded haplotypes. At the same time, the comparison highlights a clear complementarity: GFAffix effectively removes global redundancy, yet it does not resolve the alignment and interpretability issues induced by unresolved repeats. The combined PANPHORTE and GFAffix pipeline therefore yields a graph that is simultaneously more faithful to the underlying pangenome, easier to read, and better suited for downstream analyses, providing a principled and practical path toward genome graphs that are both compact and analytically robust.

Besides an optimized and faster alignment of new samples to the graph, a key consequence of topology optimization is the reduction of memory usage. By simplifying repeat-driven structures and consolidating redundant affixes, the storage required to represent a pangenome graph can be substantially decreased. This is particularly important in pangenome analysis workflows, where an attractive property of genome graphs is their ability to be iteratively augmented as new samples are aligned, progressively enriching the reference with additional variation. As a consequence, graph references naturally grow over time, making it essential to control memory occupancy without compromising the information encoded in the graph. As reported in Section 3, PANPHORTE, especially in the proposed pipeline, achieves substantial compression, with the largest gains observed for graphs with high CNV content, where explicit cyclic modeling most effectively reduces topological redundancy.

While several tools for genome graph manipulation have been proposed, none is methodologically tailored to optimize highly repetitive loci, such as CNVs, by enforcing an explicit, repeat-aware topological representation. In this context, among the most widely adopted frameworks, the vg toolkit (Garrison et al., 2018; Sirén et al., 2021) provides a comprehensive set of utilities for constructing, indexing, and augmenting genome graphs. Despite its breadth, vg does not offer dedicated functionality for topology optimization of repetitive regions. In a similar way, odgi (Guarracino et al., 2022) constitutes a valuable suite for analysis, manipulation, and visualization of variation graphs, and it includes strategies that can simplify complex regions, for instance by leveraging node-depth information. However, systematically resolving highly repetitive structures within superbubbles remains challenging, as repeat-driven variation may manifest as tangled alternative paths rather than explicit cyclic motifs. PANPHORTE is complementary to odgi in this respect: by explicitly targeting repetition-induced artefacts and refactoring them into cyclic representations, it can be used alongside odgi to improve the interpretability and compactness of particularly problematic loci. An analogous complementarity holds with GFAffix (Doerr et al., 2022), which we include in our topology-optimization pipeline. GFAffix performs general simplification by merging node affixes when overlap structure permits, reducing redundancy and streamlining the graph. Yet, because it is not designed to recognize and model repeat structure explicitly, it may leave repetitive superbubbles in suboptimal, acyclic representations (Figure 8). Incorporating PANPHORTE addresses this limitation by providing a repeat-aware optimization step; as a result, the combined pipeline yields more effective topology reduction than either approach alone, particularly in graphs enriched for repetitive variation.

## 5 Conclusion

In this work, we introduced PANPHORTE, a topology-optimization approach that identifies repeated regions misrepresented as alternative sequences in pangenome graphs and rewrites them into explicit cyclic structures. By enforcing a graph topology that faithfully captures the repetitive nature of these loci, PANPHORTE produces a more compact pangenome reference graph without sacrificing information content, enables more robust and streamlined alignment of samples carrying CNVs, and improves the visual interpretability of repeat-driven variation. We also release a C++ command-line tool implementing the PANPHORTE specifications, together with a PANPHORTE-based pipeline that further refines graph structure in regions where repeat-induced misrepresentation is not detected. Finally, through experiments on both synthetic and real datasets, we show that the resulting optimized topologies reduce memory requirements and provide tangible benefits for downstream analyses.

While PANPHORTE improves the representation of repeat-driven loci, its benefits are inherently localized to cases where repetitive structure is misrepresented within superbubbles and can be rewritten into explicit cyclic motifs. Repeats that span multiple graph regions, occur outside superbubbles, or exhibit nested or rearrangement-driven architectures may not be captured by the current detection strategy but will be explored in future developments. Additionally, integrating the PANPHORTE methodology into pangenome graph constructors would be beneficial to limit the post-processing steps to further optimize the graph. Finally, although cyclic representations facilitate the expression of CNVs, a comprehensive evaluation across additional organisms, graph constructors, and downstream tasks remains an important direction for future work. Future benchmarking on population-scale CNV datasets may further expand the characterization of PANPHORTE under diverse and heterogeneous genomic scenarios, complementing the large-scale synthetic and real-data evaluations presented in this manuscript.

## Data Availability

All the data used in this study is freely available. Bacterial data can be downloaded from the NCBI archive under these accession numbers: GCF000005845.2 (MG1655), GCF000010245.2 (W3110), GCF000019425.1 (DH10B), GCF000007445.1 (CFT073), GCF000013265.1 (UTI89), GCF000017765.1 (HS), GCF000017985.1 (REL606), GCF000008865.2 (Sakai), GCF000210475.1 (H10407), and GCF000220805.1 (C22711). MHC data can be downloaded from Zenodo at this URL: https://zenodo.org/records/6056061. Lastly, the T2T-CHM13.v2 data can be downloaded from the NCBI archive using the accession number GCA009914755.4.

## Footnotes

1
https://github.com/GenoGra/Panphorte.

2
https://github.com/GFA-spec/GFA-spec
